# Explication of Pharmacological Proficiency of Phytoconstituents from *Adansonia digitata* Bark: An *In Vitro* and *In Silico* Approaches

**DOI:** 10.1155/2024/6645824

**Published:** 2024-08-16

**Authors:** Sangavi P., Gowtham Kumar S., Nachammai KT., Chandrabose Selvaraj, Langeswaran K.

**Affiliations:** ^1^ Department of Bioinformatics Alagappa University, Karaikudi, Tamil Nadu, India; ^2^ Faculty of Allied Health Sciences Chettinad Hospital & Research Institute Chettinad Academy of Research and Education (Deemed to be University), Kelambakkam, Tamil Nadu, India; ^3^ Department of Biotechnology Alagappa University, Karaikudi, Tamil Nadu, India; ^4^ CsrDD LAB, Center for Global Health Research Saveetha Medical College Saveetha Institute of Medical and Technical Sciences, Chennai, Tamil Nadu, India; ^5^ Department of Biomedical Science Alagappa University, Karaikudi, Tamil Nadu, India

## Abstract

Compared to other drug discovery sources, traditional medicine has significantly contributed to developing innovative therapeutic molecules for preventive and curative medicine. The Baobab tree, also known as *Adansonia digitata* L., is significant in Africa due to its multitude of benefits and various parts that serve different purposes, providing economic support to rural communities. The analysis of a plant sample using Fourier transform infrared (FT-IR) spectroscopy detected multiple functional groups, such as carboxyl and aromatic groups. Additionally, gas chromatography-mass spectroscopy (GC-MS) was utilized to identify various compounds present in the sample, including tetrachloroethylene and octyl ester. The results of different assays, such as *α*-diphenyl-*β*-picrylhydrazyl (DPPH), superoxide, nitric oxide scavenging assays, and total antioxidant by thiobarbituric acid method (TBA) and ferric thiocyanate (FTC) method, demonstrated a substantial scavenging of free radicals and an effective antioxidant efficacy. The bark's antimicrobial activity was tested through agar diffusion, resulting in a range of zone of inhibition from 10.1 ± 0.36 mm to 20.85 ± 0.76 mm. The minimum inhibitory concentration (MIC) value was observed to be approximately 0.625 *µ*g/mL. The biofilm inhibition percentage ranged from 9.89% to 57.92%, with the highest percentage being 57.92%. The GC-MS and FT-IR studies revealed phytocompounds, which were then analyzed for their potential therapeutic properties. Computational studies were conducted on the phytocompounds against *Pseudomonas aeruginosa* and C2 kinase (antioxidant). The study concluded that the *Adansonia digitata* bark extract and its phytocompound have potential therapeutic efficacy against the target proteins. The best docking scores were about −7.053 kcal/mol and −7.573 kcal/mol for *Pseudomonas aeruginosa* and C2 kinase (antioxidant), respectively. The interaction patterns with the crucial amino acid residues elucidate the inhibitory efficacy of the phytocompounds.

## 1. Introduction

Medicinal herbs have been a valuable natural resource for life-threatening remedial diseases since antiquity [1]. These medicinal plants are regarded as good sources of ingredients for drug development and synthesis. Different medicinal plants can treat similar illnesses based on geographical occurrence [2]. Ethnobotanical research is required to statistically document the use of medicinal plants and their therapeutic and toxicological consequences. With 80% of all synthetic medicines produced, it is still significant today as a source for drug discovery and the primary healthcare technique for around 85% of the world's population [3]. The baobab tree, *Adansonia digitata* L., is a native of Africa. In most tropical Africa, *A. digitata* (African baobab) is one of the most significant wild food plants and a vital species with considerable ecological and social value. It is one of nine global baobab species in the *Adansonia* genus. Different solvent extracts have been shown to have therapeutic characteristics [4]. Studies have shown that extracts derived from the *Adansonia digitata* tree's bark effectively treat inflammation, diabetes mellitus, and microbial infections. The bark of this tree contains a variety of flavonoids, some of which are marketed in Europe under the name “cortex cael cedra” and used as a treatment for fever, and a substitute for cinchona bark [5].

The tree's bark contains several phytoconstituents with potential medicinal properties, including tannins, saponins, flavonoids, alkaloids, phytosterols, glycosides, and terpenoids [6]. Flavonoids are a type of polyphenolic compound that contains flavones [7]. They are composed of aromatic rings linked to three carbon atoms and cyclic structures with oxygen. They are currently used in traditional medicine to treat malaria, gastrointestinal ailments, and other diseases and infections [8]. It is a large, versatile fruit-bearing tree with over 300 traditional applications and commercial values [9]. The fruit pulp has a large amount of vitamin C, proteins, calcium, phosphate, potassium, carbs, fibers, and lipids and can be used as an appetizer or drink. Seeds are high in lysine, thiamine, calcium, and iron, including significant sodium, phosphorus, zinc, iron, magnesium, and manganese [10]. In the southern part of India (specifically Tamil Nadu), *Adansonia digitata* dotting the Ramanathapuram shoreline (Map: 13.18325184557891, 80.30669745948643) stands out with massive trunks and lush foliage. Baobabs cannot thrive in wet locations and cannot withstand heavy rain. They are only found in southern Tamil Nadu (Chennai, Madurai, and Ramanathapuram), where the climate is suitable for survival. *Adansonia digitata* shows potential as a useful source for drug development and primary healthcare approaches for an extensive percentage of the world's population when implementing consideration of traditional applications and contemporary scientific investigations on the therapeutic characteristics of the plant. However, further investigation and ethnobotanical recording need to be undertaken to grasp and exploit its promise for life-threatening restorative disorders completely.

The main aim of this study is to investigate the physicochemical characteristics and chemical composition of the bark extracts, which can be achieved using analytical tools, namely, phytochemical screening, gas chromatography-mass spectroscopy (GC-MS), and Fourier transform infrared (FT-IR) studies. This investigation's primary objective is to thoroughly examine the biological and antibacterial effectiveness of an extract obtained from *A. digitata*, also referred to as the baobab tree. We propose an integrated strategy that combines *in vitro* and *in silico* techniques to accomplish the proposed study. Our objective is to evaluate the *Adansonia digitata* ethanol extract (ADEE) efficacy against several diseases, including pathogenic microbes that form biofilms. The study seeks to ascertain the extract's effectiveness in inhibiting the biofilm formation of *Pseudomonas aeruginosa* by observing the development and morphology of biofilms under these circumstances. We aimed to obtain an in-depth comprehension of the extract's potential in different biomedical applications by carrying out precise *in vitro* and using advanced computer simulations, including molecular docking and dynamics simulation, ultimately incorporating our understanding of its therapeutic and pharmaceutical prospects.

## 2. Materials and Methods

### 2.1. Chemicals and Reagents

Mueller-Hinton agar, 2,3,5-triphenyltetrazolium chloride (TTC), Luria–Bertani broth (LB) (M1245, LOT 0000491740), dimethyl sulfoxide (DMSO), and crystal violet were purchased from Himedia (India).

### 2.2. Stem Bark Collection and Extraction

The stem bark of *Adansonia digitata* was collected from Thangachimadam, Ramanathapuram District, Tamil Nadu, India, using a sterile knife. Before being crushed into powder with a sterile pestle and mortar in a laboratory, the bark was thoroughly washed with distilled water and then air-dried in the shade for two weeks [11]. To extract secondary metabolites from the *A. digitata* bark, ethanol was utilized. The extraction process involved soaking 100 grams of powdered bark in 1000 mL of ethanol for two days at room temperature with intermittent shaking. The resulting mixture was then filtered, and the filtrate was concentrated using a rotary evaporator until a solid residue was formed. The concentrated sediment was stored at a refrigerated temperature of 4°C until required [12].

### 2.3. Assessment of Antioxidant Activity

Endogenous enzymatic antioxidants, such as superoxide dismutase (SOD), catalase (CAT), and phospholipid hydroperoxide glutathione peroxidase (GPx), serve as the first line of defense against free radicals, while nonenzymatic antioxidants, such as glutathione, vitamin C, and vitamin E, act as the second line of defense [13]. The antioxidant activity of ADEE was assessed *in vitro* through several methods, including scavenging of 2,2-diphenyl-1-picrylhydrazyl (DPPH) radical, nitric oxide scavenging, and measurement of superoxide anion scavenging activity. These tests were conducted following the methodology outlined by Kannaian et al. [14]. Additionally, the protective role of the ethanol extract in inhibiting free radical-mediated DNA-sugar damage was evaluated by estimating the total antioxidant activity of ADEE using ferric thiocyanate (FTC) and thiobarbituric acid (TBA). Vitamin C (ascorbic acid) was used as the control for this study. A brief explanation of the methodology was included in Supplementary File 1.

### 2.4. Antibacterial Activity

We evaluate the antibacterial activity of ADEE by agar well diffusion method. Bacterial isolates such as *Serratia marcescens* (MTCC97), *Escherichia coli* (MTCC739), *Pseudomonas aeruginosa* (ATCC PA01), *Klebsiella pneumonia* (MTCC1972), and *Staphylococcus aureus* (MTCC1430) were analyzed in this experiment to check the antibacterial efficacy of ADEE. Mueller-Hinton agar plates were swabbed with the bacterial cultures and incubated for 24 hrs at 37°C. Different concentrations of ADEE 25, 50, 75, and 100 *µ*g/mL were used to analyze the zone of inhibition (ZOI) [15]. After incubation, we were able to assess the remarkable antimicrobial activity of ADEE. The presence of a clear zone around the well proved its effectiveness in inhibiting microbial growth. These data highlight the potential of ADEE has potential to combat harmful pathogens. The observed zone of inhibition was measured in mm.

### 2.5. Minimum Inhibitory Concentration (MIC)

The lowest concentration of antimicrobial factor inhibits the growth of microbes after the incubation of 24 hours, referred to as minimum inhibitory concentration (MIC), and the lowest concentration required to inhibit microbial growth [16]. MIC was performed on the ADEE against bacterial isolates such as *Pseudomonas aeruginosa* (positive control and negative control) by using tetracycline as standard. The extract solution from the first test tube and 0.5 ml of nutrient broth medium (NB) were added to each tube, which was then homogenized (including sample preparation concentration) [17]. Each well was filled with dissolved 2,3,5-triphenyltetrazolium chloride (TTC), which was then incubated at 37°C for 30 min in the dark to detect respiratory activity (bacterial growth). TTC is a salt that may transform from clear to pink when digested by living bacteria. The MIC of the organism was determined to be the lowest concentration that displayed turbidity.

### 2.6. Assessment of Pellicle Formation

Glass tubes containing 5 milliliters of Luria–Bertani broth (LB) with 0.5% glucose were inoculated with test organisms, such as *Pseudomonas aeruginosa*. The treatment group was administered 1% (v/v) ethanol bark extract, while the control group received equal amounts of dimethyl sulfoxide (DMSO). The tubes were then vertically incubated at 37°C without shaking, and pellicle development at the air-liquid interface was monitored. After 72 hours, the pellicle formation was observed visually [15].

### 2.7. Biofilm Inhibition

ADEE effectively inhibits the growth of *P. aeruginosa* at the lowest concentration, as confirmed by robust validation through MIC and well diffusion methods. Furthermore, ADEE exhibits significant biofilm inhibition activity against *P. aeruginosa*, demonstrating its potential as a powerful antimicrobial agent. The day before the experiment, *P. aeruginosa* fresh culture was kept at 37°C. The overnight grown culture was taken out. Luria–Bertani broth, Miller [Himedia M1245, LOT 0000491740] was freshly prepared and sterilized. MIC endpoint of the plant extract was 0.625 *µ*g/mL. For the twofold dilution in the microtiter plates, take double the required concentration of 1.25 *µ*g/mL. All experiments were carried out in triplicate, without exception. The biofilm-inhibiting capacity of bark extracts was evaluated using the crystal violet test [18]. After 24 hours of static development at 37°C, the medium and planktonic cells were removed, for the biofilm inhibition experiment. The medium and planktonic cells were removed by rinsing each well three times with phosphate buffer. The absorbance was measured at 630 nm for planktonic cells, and the biofilm crystal violet stain was measured at 492 nm by comparing the findings with the standard (tetracycline) at 517 nm.

### 2.8. Fourier Transform Infrared Spectroscopy (FT-IR)

Fourier transform infrared (FT-IR) spectroscopy, a Thermo Nicolet 380 FT-IR spectrophotometer equipped with a KBr pellet approach with attenuated total reflection, was employed to investigate the molecular vibrations in ADEE. An appropriate quantity of the sample was deposited on the crystal surface, and the plant extract powder spectra were measured. The spectral response was recorded using OPUS software with model BRUKER-ALPHA from Germany. FT-IR spectroscopy allows the identification of characteristic functional groups present in the test samples based on the unique vibrational and rotational energies of different molecular bonds [19].

### 2.9. GC-MS (Gas Chromatography-Mass Spectroscopy)

The GC-MS (Gas Chromatography-Agilent: GC: (G3440A) 7890A. MS: 7000 Triple, Quad GC-MS) was equipped with a mass spectrometry detector. Column consisted of DB5 MS (30 mm × 0.25 mm ID × 0.25 *μ*m, composed of 5% vinyl 95% methyl polysiloxane), electron impact mode at 70 eV; helium (99.999%) was used as the carrier gas at a constant flow of 1 ml/min at injector temperature of 280°C, auxiliary temperature of 290°C, and ion-source temperature of 280°C. The oven temperature was programmed to start at 50°C (maintained for 1.0 min), then increase at a rate of 40°C/min until reaching 170°C (held for 4.0 min), and finally increase at a rate of 10°C/min until reaching 310°C (held for 10 min). The total GC running time is 32.02 min. The compounds are identified by GC-MS Library (NIST and WILEY) [20]. The ethanol extract was used for GC-MS analysis.

### 2.10. Statistical Analysis

The techniques including antioxidant activity, MIC, biofilm inhibition, and antibacterial activity were performed in triplicates. The differences between the groups were analyzed using one-way ANOVA, followed by Duncan's test for multiple comparisons (SPSS 16.0). The data analysis was done in GraphPad Prism 8.0, and the significance level was set at *p* < 0.05.

### 2.11. Computational Analysis

Furthermore, the antibacterial efficacy of the phytocompounds retrieved from bark extract was estimated using computational analysis. The studies were carried out by utilizing Schrodinger 2019-1 [21].

#### 2.11.1. Target Validation and Preparation

Biofilm and quorum sensing-mediating PqsA-modeled structure were chosen as the target of *Pseudomonas aeruginosa* by using the UniProt ID—A0A2R3IL91. Homology modeling was performed on the Prime module in Schrodinger, and the modeled structure was further validated by utilizing the Structure Analysis and Validation (SAVES) server via evaluating the Ramachandran plot (RC Plot). The plot was used to estimate the precision and stereochemistry properties of the modeled structure [22]. Additionally, *Z*-score was calculated to analyze the overall structural quality of the modeled structure, which was executed in the Protein Structure Analysis (ProSA) server [23].

By using the template structure from Protein Data Bank (PDB ID-5OE3), the structure was modeled and superimposed. The biological targets of *P. aeruginosa* and C2 kinase (antioxidant) were exposed to decipher the inhibitory effects of the phytocompounds retrieved from GC-MS analysis. The Protein Preparation Wizard is used to prepare the target structure by adding missing atoms, correcting bond orders, and adding hydrogen atoms based on protonation states. Additionally, water molecules were removed beyond 5 Å followed by adding the missing side chain [24]. The Protein Preparation Wizard automates these processes to assist in making sure that the protein structure is in an acceptable state for the next investigations, including molecular docking, molecular dynamics simulations, and structure-based drug discovery.

#### 2.11.2. Lead Optimization

The 2D structure of the chosen phytocompounds was retrieved from the PubChem database in Structured Data File (SDF) format. The LigPrep module assists in generating the possible tautomers and stereochemical forms of the compounds by using the Optimized Potentials for Liquid Simulations (OPLS_2005) force field, which helps to minimize the steric clashes of phytocompounds followed by adjusting bond angles, lengths, and torsional angles. Epik calculates protonation and tautomerization states based on a database of pKa values and rules at a pH of 7.0 ± 2.0 [25].

#### 2.11.3. Molecular Docking

Molecular docking is an extensive platform used to envisage the binding interaction between the target and the small molecule, which predicts the conformation and orientation of the ligand binding at the protein's active site [26]. The most favorable binding mode has the lowest docking score with higher precision at the binding site of the target while the docked ligand with the best score is often considered as the lead molecule for further validation. Docking was executed in extra-precision (XP) mode on the Glide module in Schrodinger [27]. Further docking was executed via the PDB entry with a cobound ligand that possesses the highest ligand similarity score to the query ligand to validate the docking process.

Partial atomic charges were considered while all other parameters were running in the default manner by adding hydrogen atoms at the pH of 7.0. Grid generation is a key phase in molecular docking studies because it determines the region in which the ligands will be investigated, and significantly improves the precision and dependability of the docking outcomes. The grid was generated over the active site residues of the target proteins with *X*, *Y*, and *Z* coordinates [28]. By carefully assessing the docking score and thoroughly analyzing the interaction patterns that indicate a significantly stronger binding affinity, we assuredly selected specific leads for further analysis.

#### 2.11.4. Molecular Mechanics with Generalized Born and Surface Area (MM/GBSA)

The energies of the free ligand and receptor are altered by their contact (GBind), and these energies also have a significant impact on the stability of the complex. Molecular mechanics with generalized born and surface area (MM/GBSA) forecasts the rate at which the interaction can be between the chosen ligand and protein, which is determined by kcal/mol [29]. MM-GBSA was performed on the Prime module in Schrodinger, which was driven by the OPLS_2005 force field in the variable solvent generalized born (VSGB) solvation model. The following equation was employed for energy calculation in MM-GBSA:

(1) $$\begin{array}{c} ∆\text{GBind}=∆\text{EMM}+∆\text{GSolv}+∆\text{GSA}, \end{array}$$

where ∆EMM represents the minimized energy of the docked complex, ∆GSolv represents GBSA and the sum of the solvation energy of the complex, and ∆GSA represents the surface area energy of the complex.

#### 2.11.5. Toxicity Prediction

Our dependence on these chemicals has reached critical levels, and failure to take decisive action now could have calamitous consequences for our health and environment. Bioavailability is a crucial factor for any oral drug candidate [30]. A high bioavailability score improves the binding selectivity profile and reduces undesirable drug effects. According to the duration and severity of the chemical exposure, this sort of exposure can be both adverse and beneficial. Therefore, it is crucial to experimentally confirm the compounds' and their combination's potential for toxicity. The selected phytocompounds were subjected to toxicity studies using ProTox-II Server [31]. The classification of the prediction tactics into various levels of toxicity, such as oral toxicity, organ toxicity (hepatotoxicity), toxicological endpoints (mutagenicity, carcinogenicity, cytotoxicity, and immunotoxicity), toxicological pathways (AOPs), and toxicology targets, offers an understanding of the possible molecular mechanisms underlying such hazardous reactions.

#### 2.11.6. Molecular Dynamics Simulation (MDS)

Molecular dynamics simulation (MDS) is a wide method used for simulating and evaluating the physical motions of atoms and molecules [32]. When the atoms and molecules are allowed to interact for a certain period, the system's dynamic progress can be observed. MDS was performed on the GROningen MAchine for Chemical Simulations (GROMACS) package for both apo and complex forms. The system was subjected to a 100 ns period with the cubic box followed by solvating the system by adding a significant amount of Na and Cl ions [33].

The whole system was minimized for structural relaxation at 50,000 steps using the Steepest descent algorithm, besides the equilibration of solvent and ions throughout the complex and apo forms under a constant number of particles, volume, and temperature (NVT) by using a method of Berendsen thermostat coupling with the coupling set at 0.1 ps at 300 K. Additionally, the constant number of particles, pressure, and temperature (NPT) ensemble was utilized to equilibrate the system using the Parrinello–Rahman pressure coupling method with the coupling constant at 2.0 ps at a pressure of 1.0 bar [34]. Finally, the final production of MD was performed for apo form at 50 ns and the generated trajectories were evaluated for further analysis to predict the stability of the modeled structure. Future studies will be focused on assessing the stability of the docked complex through MDS.

## 3. Results

### 3.1. *In Vitro* Antioxidant Studies

**Figure 1:**
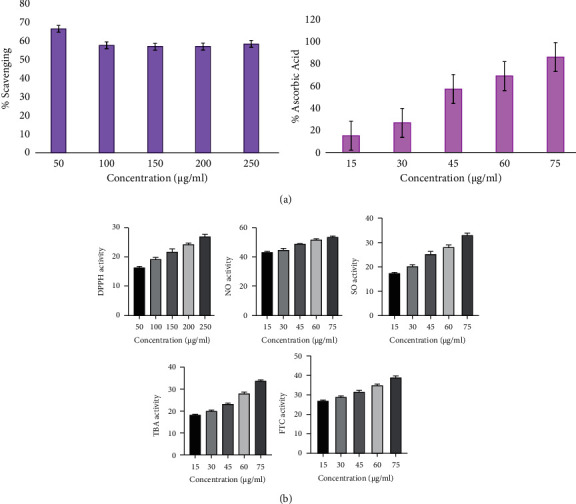
(a) Estimation of antioxidant activity of standard (ascorbic acid). (b) Estimation of antioxidant activity of *Adansonia digitata* ethanol extract (ADEE) through various methods (DPPH, NO, SO, TBA, and FTC).

In the human system, free radicals are produced, and they exist in various forms, including hydroperoxyl, peroxyl, superoxide, hydroxyl, and alkoxyl radicals. These free radicals were removed with the help of natural antioxidant enzymes. Antioxidants are the primary defense mechanism of the human body and perform lipid peroxidation inhibition. They scavenge free radicals, stopover toxic oxidation product fabrication, and uphold nutritional quality. The overall findings of antioxidant studies of *Adansonia digitata* ethanol extract (ADEE) are shown in Figure 1 and Table 1.

#### 3.1.1. 2,2-Diphenyl-1-Picrylhydrazyl (DPPH) Free Radical Trapping Activity

ADEE free radical trapping activity through the DPPH method was performed at various concentrations from 50, 100, 150, 200, and 250 *µ*g/mL. Results signify that ADEE traps more than 50% of free radicals, proving that the extract has tremendous free radical trapping activity. The results were well correlated with the standard ascorbic acid.

#### 3.1.2. Nitric Oxide Trapping Activity

ADEE was represented by nitric oxide radical strapping activity in various concentrations (15, 30, 45, 60, and 75 *µ*g/mL). These assay results displayed the significant inhibition of free radicals, comparable to the standard ascorbic acid. The outcome of this parameter suggests that the extract might be an effective peroxide inhibitor.

#### 3.1.3. Superoxide Anion-Trapping Activity

The inhibition of ADEE was achieved in a dose-dependent manner by superoxide radicals, and the resulting consequences were observed. The outcomes exhibited that the extract pointedly repressed the superoxide radicals at concentrations of 15, 30, 45, 60, and 75 *µ*g/mL. In the 75 *µ*g/mL concentration, more than 50% inhibition of superoxide was observed, and this remarkable inhibition was comparatively well with the standard ascorbic acid.

#### 3.1.4. Total Antioxidant Activity

The total antioxidant activity can be measured by two methods, FTC and TBA. The FTC method can measure the production of peroxide during the early phase of oxidation, while the TBA method can be used to measure the secondary products of oxidation, such as aldehyde and ketone.

### 3.2. Antimicrobial Activity

**Figure 2:**
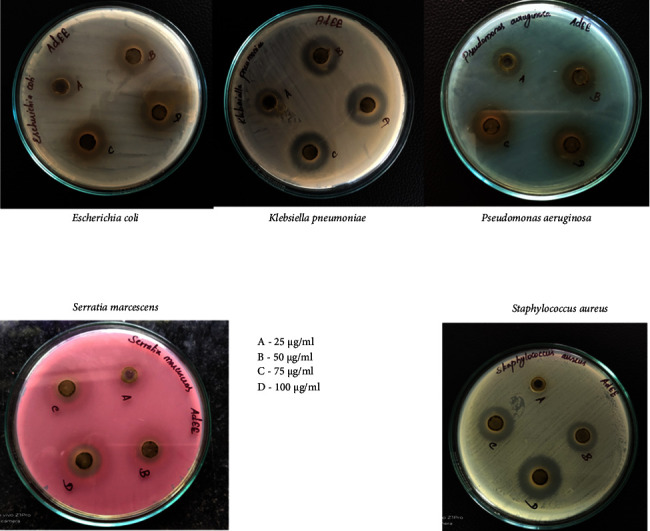
Antimicrobial activity of *Adansonia digitata* ethanol extract (ADEE) against bacterial pathogens using different concentrations.

The dose levels of 25, 50, 75, and 100 *µ*g/mL were employed to estimate the antimicrobial activity of ADEE. The antimicrobial activity was performed against *Escherichia coli*, *Klebsiella pneumoniae*, *Serratia marcescens, Pseudomonas aeruginosa*, and *Staphylococcus aureus* with the dosage of 25, 50, 75, and 100 *µ*g/mL. The zone of inhibition against bacterial pathogens through the agar well diffusion method reveals that ADEE has remarkable antibacterial efficacy, and the results are shown in Table 2 and Figure 2. The results showed that *P. aeruginosa* showed the highest zone of inhibition (20.85 ± 0.76) at 100 *µ*g/mL concentration compared with the other isolates. ADEE inhibits the growth of selected microbes with better inhibition values. The antibacterial properties of ADEE could be due to phytochemicals, such as alkaloids, tannins, saponins, flavonoids, and terpenoids, which are present in *A. digitata* bark. The differences between the groups were analyzed using one-way ANOVA, followed by Duncan's test for multiple comparisons (SPSS 16.0). Statistically significant values are represented in Table 2.

### 3.3. Minimum Inhibitory Concentration (MIC)

The minimum inhibitory concentration (MIC) is a crucial measure that determines the lowest concentration of a drug needed to effectively inhibit the growth of microorganisms. This is accurately measured by observing turbidity in a test tube, and understanding the MIC is essential in determining the drug's effectiveness. According to the well diffusion method results, ADEE may be very effective at inhibiting the growth of *P. aeruginosa*. By using twofold serial dilution techniques, the MIC was established. The MIC values and percentage yield of the ADEE against *P. aeruginosa* are shown in Supplementary Figure 1 and Table 3. Trimethyl tetrazolium chloride (TTC) is used in this technique to indicate viable microbes. Tubes 4 and 5 have the yellowish color of the broth, whereas tubes 6 and 7 have a slight reddish shade as a gradual shift into a cherry red color. Tube 5 is the minimal inhibitory action with a 0.625 *µ*g/mL concentration range. This endpoint was considered for the biofilm-inhibiting concentration range for *P. aeruginosa,* which was significantly correlated with the standard.

### 3.4. Biofilm Inhibition

Phytocompounds from plants may affect the development of biofilms by preventing the synthesis of peptidoglycan, damaging the cellular structures of microbial membranes, and affecting quorum sensing as shown in Table 4, and Supplementary Figure 2 illustrates the effects of ADEE on preventing and eradicating biofilm formation against *P. aeruginosa*. After diluting the sample, different concentrations ranging from 1.56–0.003 *µ*g/mL were added to 10 wells with positive and blank controls. Crystal violet was used to visualize the biofilm information. Well 1, with the highest concentration of extract, had the highest percentage of biofilm inhibition (57.92%), and well 10, with the lowest concentration of ADEE, had the lowest percentage of biofilm inhibition (9.89%). The absorbance of both planktonic cells and crystal violet staining was measured at 630 nm and 492 nm. The observed results were significantly correlated with the standard (tetracycline) values.

### 3.5. Assessment of Pellicle Formation

**Figure 3:**
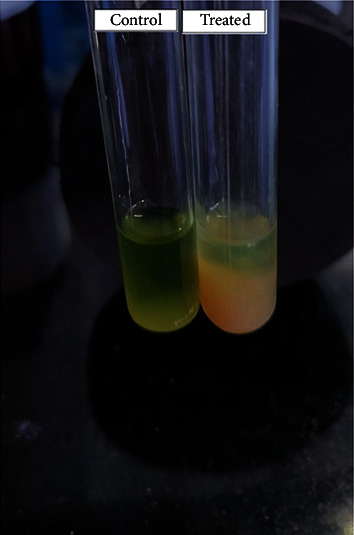
Inhibition of pellicle formation by ADEE.

PAO1 gene in *Pseudomonas aeruginosa* coded for pellicle production (a thin biofilm around cells that forms at the air-liquid interface of a standing liquid culture). Inhibiting the formation of pellicles is necessary because it can aid in preventing the development of biofilms, which are surface-associated microbial populations enclosed in an extracellular matrix in *P. aeruginosa,* which could diminish severe infections. The treatment of ADEE (35 *µ*g/mL) notably reduced pellicle formation at 12 *µ*g/mL markedly condensed pellicle development was experimental in PAO1 (Figure 3).

### 3.6. Fourier Transform Infrared Spectroscopy

**Figure 4:**
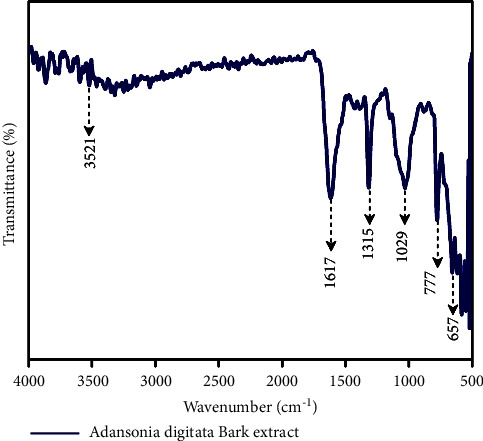
Interpretation of FT-IR spectra of the ethanol extract obtained from *Adansonia digitata* bark.

The FT-IR analysis revealed ADEE's peak values and functional groups' presence in the bark ethanolic extract. The various functional groups observed in the ADEE indicate the presence of ketone, carboxylic acid, silicon, phosphate, alkanes, chloro, bromo, and hydroxy compounds, and the plot is illustrated in Supplementary Table 1 and Figure 4. The presence of functional constituents in the given extract was analyzed flawlessly using the FT-IR spectrum, which may support the extract's biomedical properties and therapeutic efficacy.

### 3.7. GC-MS

GC-MS results of ADEE exhibited that different phytocompounds were identified. The report showed five major peaks (5-octadecene; (E)-pentanoic acid, 5-hydroxy-, 2,4-di-t-butyl vinyl esters; 1-nonadecene; 5-eicosene; 1-octadecanol) from the ethanol extracts. The best hits were selected based on their biological properties, therapeutic efficacy, and peak range. ADEE showed better phytocompounds, and these extracts were subjected to further investigations. Results and findings from ADEE are shown in Supplementary Table 2, and the chromatogram is displayed in Supplementary Figure 3.

### 3.8. Target Validation

**Figure 5:**
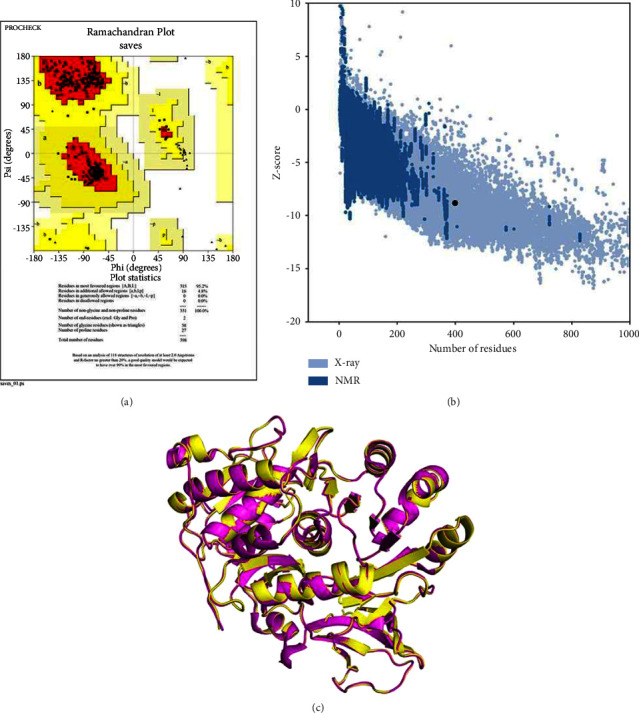
Validating modeled structures can be done effectively through Ramachandran plot and *Z*-score analysis. (a) Ramachandran plot. (b) *Z*-score −8.79. (c) Superimposition of modeled and template structure. Yellow modeled structure, magenta template (PDB ID-5OE3).

The pseudomonas quinolone signal (PqsA) structure was modeled using the template while the superimposed structure was visualized using PyMOL. Furthermore, the model was validated using the Ramachandran plot to gain insights into the modeled structure. The regions significantly occupied are the alpha-helices and beta-sheets, which are typical secondary structures in proteins. The Ramachandran plot depicted that 95.2% region is occupied by the most favored region while the disallowed region was found to be 0.0%, which is shown in Figure 5. Additionally, the *Z*-score value was found to be −8.79 and the modeled structure was superimposed with the template, resulting in an RMSD value of approximately 0.23 Å.

### 3.9. Apo Dynamics of the Modeled Structure

**Figure 6:**
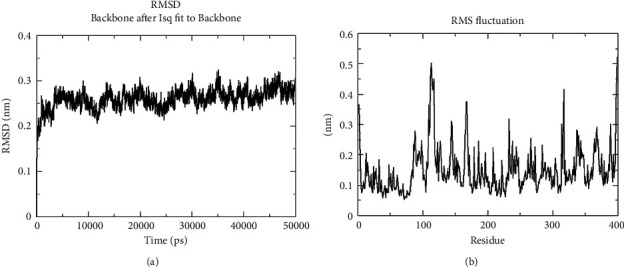
Molecular dynamics simulation of modeled structure: (a) RMSD and (b) RMSF plots, which decipher the structural stability.

Apo dynamics aid in understanding the role of protein performance in its natural environment, regulation, and interaction with other molecules. The development of more potent therapeutics is rendered feasible by the utilization of dynamic regions in the apo form that regulates the generation of small molecules or drugs that target certain protein conformations or states. The modeled structure of *P. aeruginosa* was subjected to molecular dynamics simulation by utilizing the GROMACS package employing the GROMOS96 43a1 force field.  Figure 6 illustrates the root-mean-square deviation (RMSD) and root-mean-square fluctuation (RMSF) plot, which deciphers the overall structural stability of the modeled structure. The average RMSD value was observed at 0.25−0.3 nm, which was under the threshold, and initially, mild deviation was observed at 0.25 nm and reached the maximum at 0.3 nm. Interestingly, the RMSD plot interprets the stability throughout the simulation period without any aggressive deviation while the average fluctuation rate was observed at 0.15–0.25 nm. The maximum fluctuation rate was detected on 100–125 residue at 0.5 nm, which occurred at the loop region and did not cause any structural changes or affect the structural stability of the complex. The overall MDS findings concluded that the modeled structure was stable without much deviation and fluctuation. Hence, it was considered for further analysis.

### 3.10. Molecular Docking

#### 3.10.1. Interaction between *Pseudomonas aeruginosa* Phytocompounds

**Figure 7:**
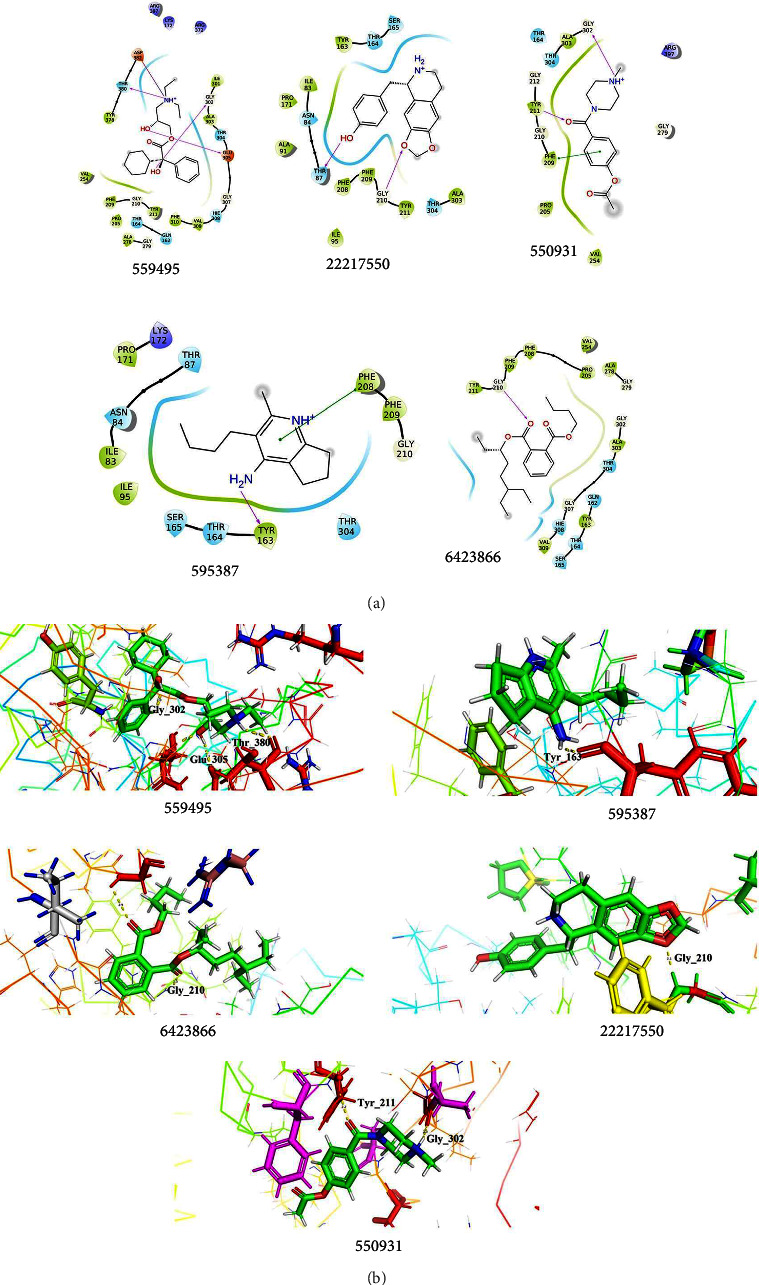
Docking interpretation of phytocompounds with the modeled structure of *Pseudomonas aeruginosa*. (a) 2D interpretation of the target interacted with the phytocompounds. (b) The 3D pattern of the docked complexes showed the interaction between the target and the lead phytocompounds.

Identifying novel targets for *Pseudomonas aeruginosa* infection management may be possible by better figuring out the quorum sensing processes that restrict biofilm formation. From the *in vitro* experiments, *P. aeruginosa* showed the highest inhibitory activity when compared with the other isolates, while the biofilm formation was inhibited by the bark extract. The chosen phytocompounds were docked with the modeled structure of *P. aeruginosa* (pqsA) using the template PDB ID–5OE3. Out of selected phytocompounds, five docked complexes (559495, 22217550, 550931, 595387, and 6423866), which are illustrated in Figure 7 and tabulated in Table 5, showed a better docking score of about −7.053, 6.743, −5.745, −5.149, and −4.963 kcal/mol, respectively, while the interacting residues formed hydrogen bond, salt bridge, and Pi-Pi interaction with the key residues. Three hydrogen bond interactions were noted with Glu_305 (2.14 Å), Gly_302 (2.03 Å), and Thr_380 (2.11 Å) via the OH group and amine group, which facilitates the structural stability of the complex, while the OH group interprets as both donor and acceptor in a hydrogen bond; hence, the complex maintained the stability via the formation of hydrogen bonds. Asp_382 interacted with NH⁺ and formed salt bridge interaction at the distance of 4.48 Å, which possesses a carboxyl group in its side chain that tends to lose a proton and deteriorate negatively charged, allowing it to interact form salt bridge interaction and is involved in protein folding and stabilize the native conformation of the target. 22217550 complexes showed Thr_87 (1.99 Å) and Gly_210 (2.06 Å) formed hydrogen bond interaction with OH and oxygen groups, which paves the way to stable interaction between the target and the compounds. Two hydrogen bonds and one Pi-Pi interaction were noted in 550931 complexes among Gly_302 (2.29 Å), Tyr_211 (2.21 Å), and Phe_209 (4.38 Å). The potential for the phytocompounds to be effective as potent ligands for the target protein has been demonstrated by docking investigations that indicate significant scores and interactions with key amino acid residues. This insight can assist in researching and developing novel drugs since it identifies the phytocompounds that are most probable to bind to the target and perhaps modify its activity.

#### 3.10.2. Interaction of 2OXX_Phytocompounds

Protein kinase CK2 docked with the chosen phytocompounds showed a docking score of about −7.573 kcal/mol (559495), −7.462 kcal/mol (22217550), −6.710 kcal/mol (106994), −6.130 kcal/mol (550931), and −5.960 kcal/mol (20393), respectively. By exploring the interactions of 2OXX_559495, it was noted that the hydroxyl group and amine group made interaction with Hie_160 (2.21 Å) and Asp_175 (1.66 Å) and formed hydrogen bond interaction, while salt bridge interaction was noted at Asp_175 (1.66 Å) to interact with the amine group. Second, the 2OXX_22217550 complex exhibited two hydrogen bond interactions with Tyr_115 (2.62 Å) and Val_45 (2.16 Å), which were found to be in contact with the hydroxyl and amine groups.

**Figure 8:**
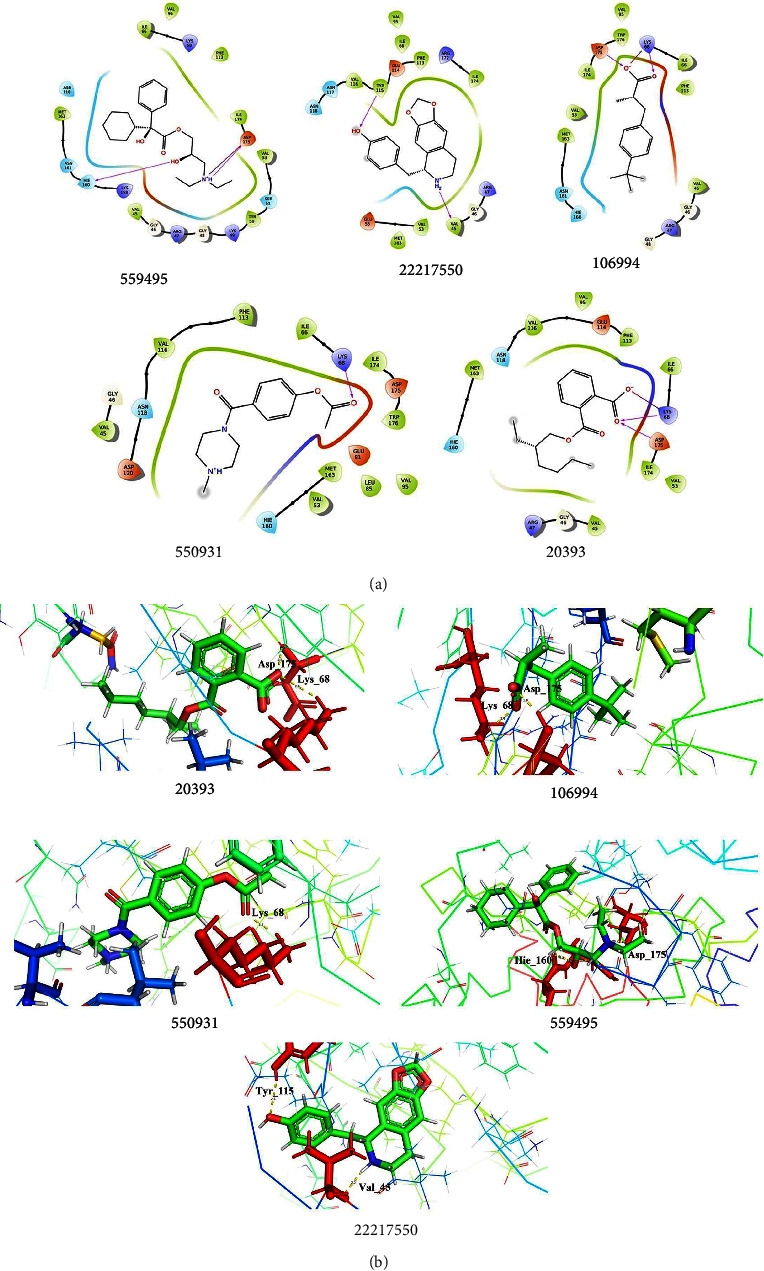
Docking analysis of 2OXX_Phytocompounds. (a) 2D interaction profiles of 2OXX interacted with the lead phytocompounds. (b) 3D interaction profiles of the docked complexes showed hydrogen bonds and other interactions.

The complex 2OXX_106994 deciphers the hydrogen bond formation with Asp_175 and Lys_68 at the distance of 1.85 Å and 1.95 Å, while Lys_68 (4.53 Å) also interacts with the oxygen group by forming salt bridge interaction, which favors the stability, conformational rigidity, and folding of the target structure. The other two complexes 550931 and 20393 interacted through hydrogen bonds with Lys_68 (2.05 Å and 2.60 Å) and Asp_175 (2.51 Å), and those interactions were found to be made with the oxygen group. The overall docking results depicted in Figure 8 and Table 6 concluded that the resultant phytocompounds showed good docking scores interacting with the crucial amino acid residues within the active site of the target. The highest docking score suggests a strong or favorable binding affinity subsidizing protein-ligand stability. Hence, the study concluded that the phytocompounds docked with the modeled structure of *P. aeruginosa* and 2OXX were found to be potential or effective ligands against the target. Both *in vitro* and *in silico* analyses suggest that the phytocompounds in *A. digitata* extract exhibited their potent activity as drug candidates and pharmacological efficacies.

### 3.11. MM-GBSA

The docked and binding efficacy of the complex was further validated by binding free energy calculation by implementing MM-GBSA.  Table 7 demonstrates the calculated energy values of the docked complex of the modeled structure and 2OXX_Complex. The molecule that needed the least energy to fit comfortably inside the protein's active region is considered to be the best for inhibition or activation; additionally, the stability of the docked complex is significantly influenced by MM-GBSA. Overall, negative energies signify a higher level of stability. The calculated MM-GBSA scores of the modeled structure with the phytocompounds were identified as 559495 (−47.40 kcal/mol), 22217550 (−43.91 kcal/mol), 595387 (−39.87 kcal/mol), 6423866 (−50.42 kcal/mol), and 550931 (−40.55 kcal/mol), respectively.

Followed by 2OXX complexes showed better binding free energy, which were well interrelated with the docking scores. The interactions such as hydrogen bonds, salt bridges, and Pi interactions favored the stability of the docked complexes, whereas the MGBSA scores decipher the precise assessment of bonding affinities of the complex. The calculated MM-GBSA values of 2OXX with the phytocompounds have the average value observed between −40.67 and −50 kcal/mol, respectively. In conclusion, the MM/GBSA estimation defined the binding affinities of the highest and lowest compounds in the precise positions as docking energy although there were minor modifications in the order for compounds holding intermediate docking energy. Future studies will be enabled to further validate the compound stability by MDS, and we have taken all of the chosen complexes.

### 3.12. Toxicity Prediction

We determined the toxicity parameters of the phytocompounds using ProTox-II Server (Table 8). The GHS guidelines classify substances into six groups, with the highest group having the lowest likelihood of being dangerous or damaging. Classes 1 (LD50 ≤ 5) and 2 (5 < LD50 ≤ 50) indicate that they are lethal and very certainly will result in death. In this analysis, it was determined that none of the chemicals belonged to classes 1 or 2. Class 3 indicates potential toxicity hazards from oral exposure (50 < LD50 ≤ 300), and compound_595387 belongs to Class 3. Class 4 (300 < LD50 ≤ 2000) classifies compounds that are most likely to be dangerous when consumed; in this case, 22217550, 559476, 6423866, 550931, 559495, and 20393 were found to be in Class 4. Compound_106994 falls in Class 6 (LD50 > 5000), which determines the nontoxic nature of the phytocompound. The toxicity of phytocompounds was assessed using toxicity criteria like mutagenicity, carcinogenicity, hepatotoxicity, immunogenicity, etc., which were all found to be inactive in most of the phytocompounds.

## 4. Discussion

Plant-derived bioactive substances known as phytochemicals have been associated with reducing chronic degenerative diseases in humans [35]. *Nigella sativa* extract contains thymoquinone, which can effectively upregulate and downregulate several biomarker signaling pathways, thereby providing a potential therapeutic target for TNBC [36]. Numerous studies have documented the potential of isoliquiritigenin, derived from *Glycyrrhiza glabra*, to impede the expression of the NF-*κ*B pathway. Additionally, it could lessen the extent of myocardial infarctions, lower the production of proinflammatory markers, and enhance heart function in mice [37]. Several studies postulated that quercetin might prevent and treat COVID-19 due to its potent scavenger and anti-inflammatory properties. Fifty COVID-19-infected individuals received a daily dosage of 1,000 mg of quercetin as part of a randomized clinical study [38]. Various fruits contain Fisetin, which may be effective in preventing traumatic brain damage, schizophrenia, depressive disorders, stroke, and neuropathy associated with diabetes [39]. Withaferin A has demonstrated anticancer activity in several cancer cell types, including multiple myeloma, neurological tumors, leukemia, and an aggressive form of ovarian, breast, and neck cancer [40]. One of the approaches for determining the quality of herbal medication is phytochemical examination [41]. The ethanol extract of *A. digitata* (ADEE) bark revealed the presence of flavonoids, phenols, triterpenoids, saponins, and other compounds. Previously, ADEE was reported on several disorders, such as cancer, renal, and diabetes. The defense system against oxidation can remove reactive oxygen species (ROS), which have a crucial role in starting lipid peroxidation [42]. DPPH (1, 1-diphenyl-2-picrylhydrazyl) radical scavenging activity measures antioxidants hydrogen-donating ability, and the relative decrease in DPPH absorbance when it reacts with the antioxidant measures its activity [43]. The ethanol extract demonstrated a concentration-dependent increase in free radical trapping in the current investigation. *In vitro*, the ethanol extract lowered NO production in a concentration-dependent way. A superoxide anion is a reduced form of molecular oxygen formed by receiving one electron. According to the findings of this investigation, the ethanol extract has high superoxide quenching action. This could be related to the phenol level of the ethanol extract. Free radicals can harm macromolecules in cells like DNA, protein, and membrane lipids. Lipid peroxidation refers to the reaction of oxidative deterioration of polyunsaturated lipids. Peroxidation involves the direct reaction of oxygen and lipids to form radical intermediates and produces semiconstant peroxidases, damaging the enzymes, nucleic acids, membranes, and proteins [44]. The ethanol extract of bark prevents DNA damage by quenching free radicals in the current study. The ferric thiocyanate (FTC) method was used to calculate the total antioxidant activity of bark and was compared to the thiobarbituric acid (TBA) method. The antioxidant activity of ADEE was relatively high in this study. These points to the existence of antioxidant-active phytochemical elements like ascorbic acid and carotenoids both detected in ADEE.

Although there are multiple antibacterial agents to choose from, they often suffer from several disadvantages, such as low solubility, limited stability, high expense, poor bioavailability, challenges in accessing the site of action, frequent administration, significant side effects, and potential toxicity [45]. At different concentrations, the antimicrobial activity of ADEE against *Klebsiella pneumoniae, Staphylococcus aureus, Escherichia coli, Serratia marcescens,* and *Pseudomonas aeruginosa* was observed. ADEE inhibited the growth of the test organisms at a concentration of 75 *µ*g/mL and 100 *µ*g/mL with a slight zone of inhibition at 25 *µ*g/mL and 50 *µ*g/mL. This antibacterial activity shown by ADEE indicated the active compound(s) extracted by the cold maceration method. *P. aeruginosa* was used to test pellicle production in glass tubes containing LB broth at two different temperatures (25°C and 35°C). After 24 hours, a thin pellicle formed on the media surface, followed by an opaque, strong, thick pellicle covering the whole media. Aerobic bacteria require this contact because they acquire oxygen from the air and nutrients from the liquid media [46].

Investigators distinguish closely related flora and fauna by applying the powerful tool FT-IR spectrum [47]. In this present study, functional groups and bioactive compounds in the plant extract were revealed using FT-IR analysis. Revealing phytocompounds from the resultant GC-MS and FT-IR studies paved the way for analyzing the potential therapeutic phytocompounds. The presence of functional groups such as ketones, carboxylic acid, alkanes, and silicon plays a key role in various medicinal ailments of *A. digitata*, in which silicon contributes to the proper functioning and prevents the toughening of arteries and veins. On the other hand, the contribution of carboxylic acid and hydroxyl groups leads to the proper functioning and metabolism, and the presence of such functional groups is considered a remarkable therapeutic ailment. Furthermore, computational studies revealed the phytochemicals' potency against the virulence factors of the targeted isolates and their antioxidant enzyme activity in this study. Previous studies reported that the phytocompounds such as apigenin and quercetin isolated from the bark extract possess antiplasmodial activity [48], whereas the interaction of quercetin and apigenin inhibit the *β*-hematin formation, which was revealed through the highest binding affinity and interaction of hydrogen bond formation.

To ascertain the strength of the interaction and determine the optimal orientation of the interaction between molecules present in *Adansonia digitata* and vascular endothelial growth factor receptor 2, which would result in a complex with protein with the least energy, molecular docking experiments have been carried out. All the compounds have demonstrated the suppression of hepatobiliary cancer-causing vascular endothelial growth factor 2 (VEGF2) [49]. In this study, the lead phytocompounds showed better binding energy and interaction with the key amino acid residues easing the inhibitory efficacy against the target protein modeled pqsA and 2OXX. Residues such as Gly_302, Tyr_163, Gly_210, and Thr_87 from the modeled structure interacted with the hydroxyl and amine groups, facilitating the stronger interaction and structural stability of the docked complex. Results obtained from 2OXX interact with the phytocompounds explicitly showing its potent efficacy by forming hydrogen bonds and Pi-Pi interaction with the crucial residues with better binding affinity values. The presence of ketone, carboxylic acid, alkanes, and polyhydroxy functional groups present in *A. digitata* illustrates the biological and pharmacological efficacy, while those functional groups play a crucial role in interacting with the key residues of the targets.

Research on the possibility of using extracts of plants to treat degenerative disorders that persist over time is rapidly expanding. Given the increasing frequency of these illnesses, the encouraging potential provided by plant extracts provides a glimpse of hope for people afflicted with these devastating conditions. The future perspective of this study deals with the phytocompounds from *Adansonia digitata* that could be examined against chronic diseases including cancer. The overall study concluded that the ADEE showed stronger inhibition efficacy against the selected isolates through MIC and biofilm inhibition, while the studies were further evaluated by *in silico* approaches, which paves the way to conclude the potent drug-likening property of the phytocompounds present in *Adansonia digitata*.

### 4.1. Strengths and Limitations of the Study

This investigation employs a multidisciplinary methodology and acknowledges the importance of conventional medicine as the origin of novel medicinal compounds. It pays attention to the commercial benefits that baobab trees offer to rural areas. Despite their value, the computational investigations rely on simulations and predictions. Therefore, to confirm their accuracy and applicability, *in vitro* and *in vivo* research types are required. Furthermore, more investigation needs to be done, including clinical studies and quality control procedures, to verify its safety and efficacy for certain medicinal applications. A more comprehensive perspective can be provided by taking into consideration the cultural and economic facets of traditional medicine. According to the study's findings, *Adansonia digitata* bark extract and its phytocompounds may be useful as medicines, especially against *Pseudomonas aeruginosa* and C2 kinase.

## 5. Conclusion

*Adansonia digitata* has been considered a versatile tree species used as a remedy against various illnesses, fodder, valued nutritional food, and regarded as raw material for making valuable items. The outcome of the phytochemical screening analysis conducted during this investigation irrefutably confirms the presence of numerous phytoconstituents, such as alkaloid, saponin, flavonoid, steroid, tannin, and terpenoids, in the stem bark extract. GC-MS and FT-IR spectroscopic analysis results show that the extract has numerous functional groups that contribute to its potential as a therapeutic. ADEE's ability to suppress pathogenic bacteria and its therapeutic efficiency, especially in preventing the formation of pellicles and inhibiting biofilms, are probably due to a variety of secondary metabolites found in the bark. At lower concentrations, ADEE revealed more than 80% inhibition of *P. aeruginosa* biofilm formation while the observed MIC value of about 0.625 *µ*g/mL. Utilizing the application of computational techniques to analyze the phytocompounds found in the bark of *A. digitata*, we have shown that this natural component possesses remarkable binding ability and constant stability against selected receptors. As a result, we can assert with certainty that the bark of *A. digitata* is a powerful source of therapeutic targets and therapeutic advantages, efficiently alleviating a range of diseases. Additional investigation of these phytocompounds and their specific measures may provide insightful information for conventional and modern applications.

## Figures and Tables

**Table 1:** (a) Antioxidant activity of standard (ascorbic acid) DPPH. (b) Antioxidant activity of *Adansonia digitata* ethanol extract (ADEE) DPPH.

(a)				
S. no	Standard (ascorbic acid) (*μ*g/mL)	% of DPPH scavenging		

1	50	66.25		
2	100	57.35		
3	150	56.53		
4	200	56.77		
5	250	58.23		

S. no	Standard (ascorbic acid) (*μ*g/mL)	% of ascorbic acid (NO, SO, TBA, FTC)		

1	15	14.7		
2	30	26.2		
3	45	56.4		
4	60	68.4		
5	75	85.5		

(b)				
Ethanol extract (*μ*g/mL)	DPPH activity			

50	16.3 ± 0.24			
100	19.4 ± 0.6			
150	21.8 ± 0.7			
200	24.32 ± 0.6			
250	27.04 ± 0.7			

Ethanol extract (*μ*g/mL)	NO activity	SO activity	TBA activity	FTC activity

15	43.23 ± 0.63	17.33 ± 0.11	18.34 ± 0.3	26.68 ± 0.8
30	44.59 ± 1.10	20.51 ± 0.6	20.20 ± 0.3	28.74 ± 0.33
45	49.08 ± 0.6	24.33 ± 0.6	23.42 ± 0.3	31.39 ± 0.8
60	51.90 ± 0.5	28.45 ± 0.8	26.97 ± 0.6	34.82 ± 0.8
75	53.78 ± 0.7	33.10 ± 0.7	33.74 ± 0.8	39.11 ± 0.23

Values are expressed as mean ± SD for triplicates.

**Table 2:** Antibacterial activity of *Adansonia digitata* ethanol extract (ADEE) through the agar well diffusion method.

Concentrations (*μ*g/mL)	Zone of inhibition (mm)				
	*Klebsiella pneumoniae*	*Staphylococcus aureus*	*Escherichia coli*	*Serratia marcescens*	*Pseudomonas aeruginosa*
25	11.27 ± 0.8^a^	10.1 ± 0.4^b^	12.93 ± 0.9^c^	13.43 ± 0.4^c^	14.9 ± 0.17^d^
50	16.56 ± 0.4^a^	15.53 ± 0.5^b^	16.8 ± 0.34^a^	14.67 ± 0.4^c^	16.6 ± 0.4^a^
75	17.8 ± 0.72^a^	18.9 ± 0.17^b^	17.93 ± 0.3^a^	16.73 ± 0.3^c^	18.83 ± 0.8^b^
100	20.57 ± 0.51^a^	20.2 ± 0.9^bc^	20.03 ± 0.5^b^	18.7 ± 0.4^c^	20.85 ± 0.8^bc^

Values represented in the table are means of triplicate readings and standard error of the mean of zone of inhibition of isolates. a, b, c, and d likely represent statistically significant differences between the zones of inhibition, which indicate the effectiveness of the extract against different bacteria at varying concentrations.

**Table 3:** Different concentrations of ADEE used for the MIC determination.

Tube number/label	Concentration (*μ*g/mL) of compound	Observed result	
		Antibiotic (tetracycline)	ADEE
1	10 *μ*g/mL	MIC	MIC
2	5	MIC	MIC
3	2.5	MIC	MIC
4	1.25	MIC	MIC
**5**	**0.625**	**MIC**	**MIC**
6	0.3125	MIC	Growth
7	0.15625	Growth	Growth
8	0.078125	Growth	Growth
9	0.0390625	Growth	Growth
10	0.01953125	Growth	Growth
11-Positive control	NIL	Growth	Growth
12-Blank/sterility control	NIL	—	—

The value 0.625 represents the minimum inhibitory concentration (MIC) of the antibiotic tetracycline, indicating the lowest concentration (10 *μ*g/mL) of the aqueous extract of ADEE at which tetracycline successfully inhibited bacterial growth. This MIC value highlights tetracycline's potency against the tested bacteria, even at a relatively low concentration when combined with ADEE.

**Table 4:** Biofilm inhibition percentage of ADEE.

Well no.	ADEE concentration (*μ*g/mL)	OD reading of planktonic cells (630 nm)	OD reading of biofilm crystal violet stain (492 nm)	Percentage of biofilm inhibition (ADEE) (%)	Percentage of biofilm inhibition (standard) (%)
1	1.56	0.281	0.319	57.92	92.74
2	0.78	0.575	0.460	39.31	90.06
3	0.39	0.210	0.421	44.46	89.34
4	0.19	0.283	0.653	41.69	88.62
5	0.09	0.643	0.442	27.44	84.95
6	0.04	0.283	0.550	17.94	76.0
7	0.02	0.474	0.622	13.85	73.12
8	0.01	0.651	0.714	5.8	68.06
9	0.006	0.340	0.676	10.82	70.91
10	0.003	0.281	0.683	9.89	66.64
11	CONTROL	0.307	0.758	N.A.	N.A.
12	BLANK	0.068	0.011	N.A.	N.A.

**Table 5:** Docking interpretation of modeled structure with the phytocompounds.

Compound ID	2D structure	Docking score (kcal/mol)	Glide energy	Interacting residues
559495	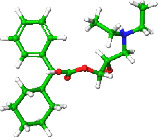	−7.053	−40.623	HB-Glu_305 (2.14 Å), Gly_302 (2.03 Å), Thr_380 (2.11 Å) SB-Asp_382 (4.48 Å)

22217550	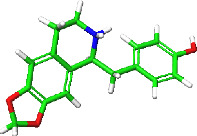	−6.743	−36.821	HB-Thr_87 (1.99 Å), Gly_210 (2.06 Å)

550931	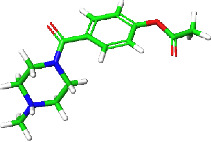	−5.745	−30.403	HB-Gly_302 (2.29 Å), Tyr_211 (2.21 Å) Pi-Pi-Phe_209 (4.38 Å)

595387	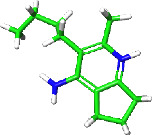	−5.149	−24.906	HB-Tyr_163 (2.23 Å) Pi-Pi-Phe_208 (4.12 Å)

6423866	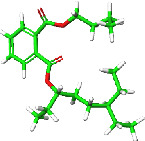	−4.963	−20.724	HB-Gly_210 (2.15 Å)

**Table 6:** Docking interpretation of 2OXX with the phytocompounds.

Compound ID	2D structure	Docking score (kcal/mol)	Glide energy	Interacting residues
559495	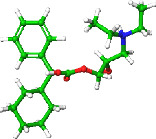	−7.573	−40.340	HB-Hie_160 (2.21 Å), Asp_175 (1.66 Å) SB-Asp_175 (1.66 Å)

22217550	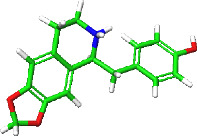	−7.462	−35.992	HB-Tyr_115 (2.62 Å), Val_45 (2.16 Å)

106994	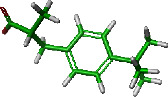	−6.710	−21.187	HB-Asp_175 (1.85 Å), Lys_68 (1.95 Å) SB - Lys_68 (4.53 Å)

550931	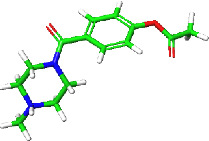	−6.130	−33.771	HB-Lys_68 (2.05 Å)

20393	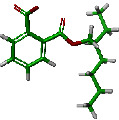	−5.960	−30.555	HB–Lys_68 (2.60 Å), Asp_175 (2.51 Å) SB-Lys_68 (3.66 Å)

**Table 7:** Binding free energy of the docked complexes computed by MM/GBSA.

Modeled structure		PDB ID: 2OXX	
Compound ID	Δ*G* bind (kcal/mol)	Compound ID	Δ*G* bind (kcal/mol)
559495	−47.40	559495	−48.13
22217550	−43.91	22217550	−51.87
550931	−40.55	106994	−41.43
595387	−39.87	550931	−39.65
6423866	−50.42	20393	−37.32

**Table 8:** Toxicity prediction of the phytocompounds using ProTox-II server.

Compounds	LD50 (mg/kg)	Toxicity class	Hepatotoxicity	Carcinogenicity	Immunotoxicity	Mutagenicity	Cytotoxicity
22217550	940	4	Inactive	Inactive	Less active	Inactive	Inactive
559476	1600	4	Inactive	Inactive	Less active	Less active	Inactive
6423866	1340	4	Inactive	Less active	Inactive	Inactive	Inactive
550931	2000	4	Inactive	Inactive	Inactive	Inactive	Inactive
595387	68	3	Inactive	Inactive	Inactive	Inactive	Inactive
559495	480	4	Inactive	Inactive	Inactive	Inactive	Inactive
106994	15000	6	Inactive	Inactive	Inactive	Inactive	Inactive
20393	1340	4	Inactive	Less active	Inactive	Inactive	Inactive

## Data Availability

Some of the data that support the findings of this research are included in the supplementary information accompanying this article. The remaining datasets used to generate or analyze during this study can be obtained from the corresponding author upon reasonable request.

## Additional Points

*Use of AI Software or Tools*. None of the AI sources were used in this manuscript.

## Supplementary Materials

Supplementary Materials Supplementary Figure 1: minimum inhibitory concentration (MIC) of ADEE. Supplementary Figure 2: biofilm inhibiting activity of ADEE. Supplementary Figure 3: GC-MS chromatogram of ethanol extract of *Adansonia digitata* bark. Supplementary Table 1: FTIR interpretation of phytocompounds of ethanol extract of *Adansonia digitata* bark. Supplementary Table 2: GC-MS chromatogram of ethanol extract of *Adansonia digitata* bark.
