# Supplementary material for: Explication of Pharmacological Proficiency of Phytoconstituents from Adansonia digitata Bark: An In Vitro and In Silico Approaches
Source: Scientifica (Cairo). 2024 Aug 16;2024:6645824. doi: 10.1155/2024/6645824 (PMC11343629; doi:10.1155/2024/6645824)
Supplement: Supplementary Materials — Supplementary Figure 1: minimum inhibitory concentration (MIC) of ADEE. Supplementary Figure 2: biofilm inhibiting activity of ADEE. Supplementary Figure 3: GC-MS chromatogram of ethanol extract of Adansonia digitata bark. Supplementary Table 1: FTIR interpretation of phytocompounds of ethanol extract of Adansonia digitata bark. Supplementary Table 2: GC-MS chromatogram of ethanol extract of Adansonia digitata bark. [file 6645824.f1.docx]

**Supplementary Figure 1:** **Minimum Inhibitory Concentration (MIC) of ADEE**


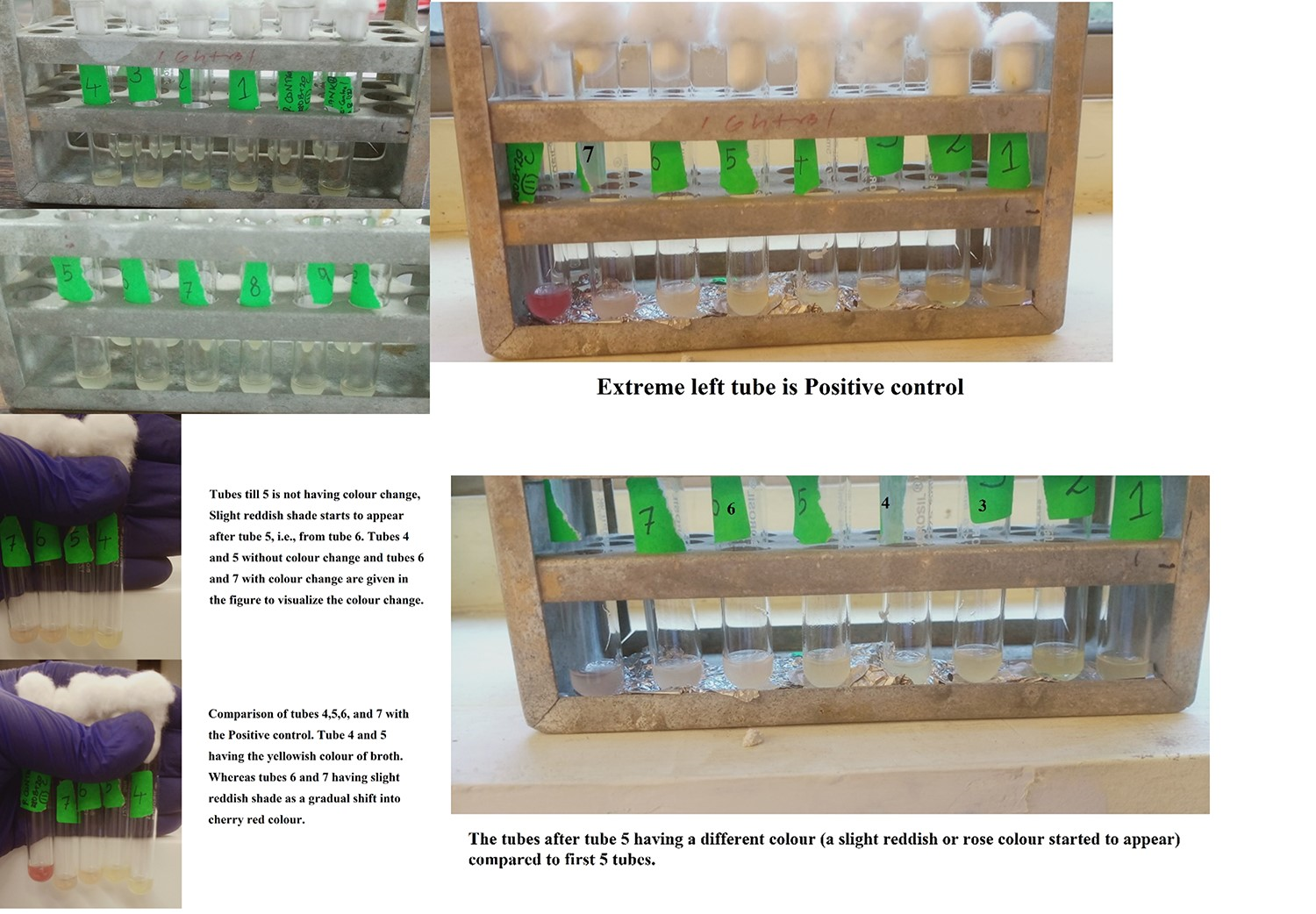


**Culture tubes were taken out after 24 hrs incubation and after the treatment of TTC**

**Supplementary Figure 2: Biofilm inhibiting activity of ADEE**


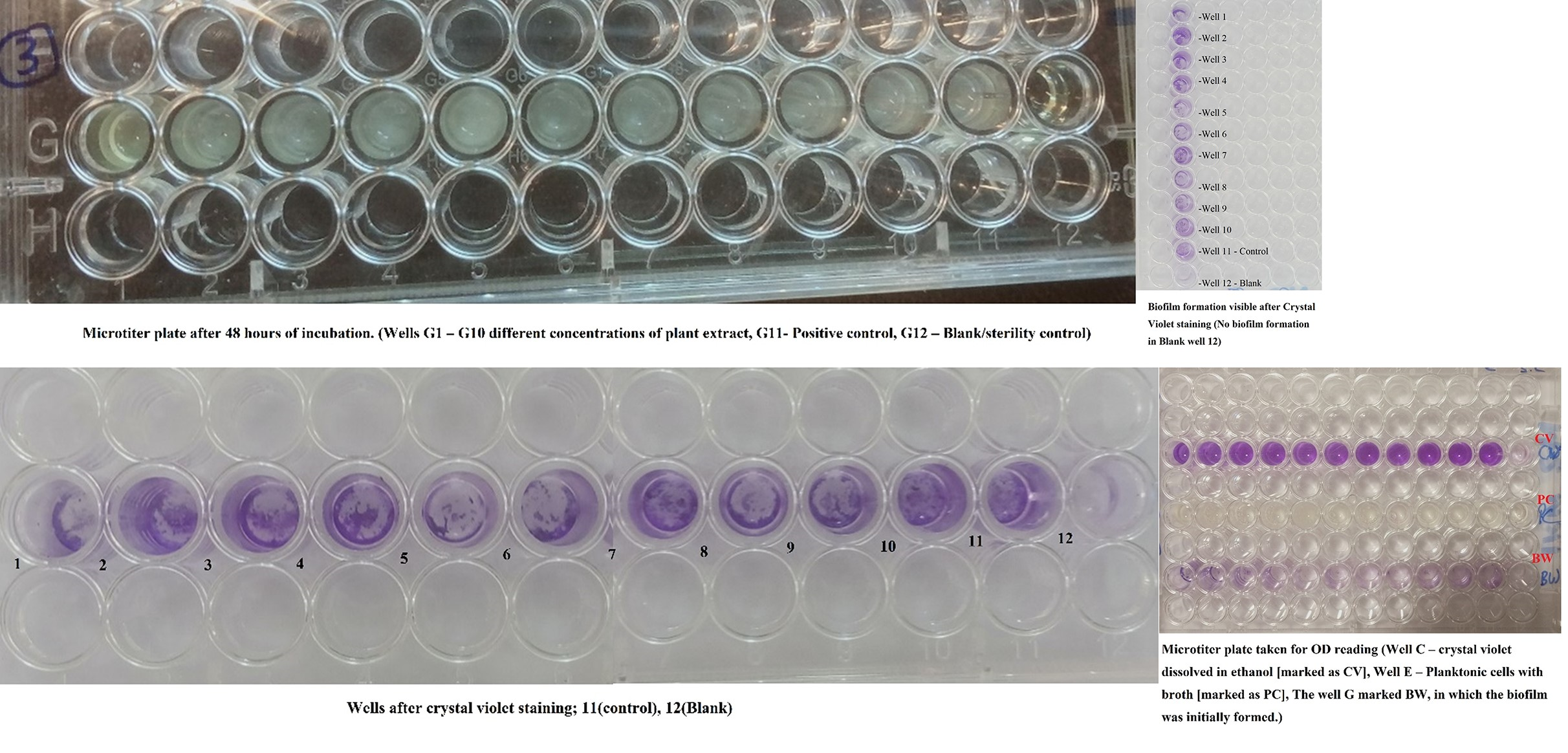


**Supplementary Figure 3: GC-MS Chromatogram of ethanol extract of *Adansonia digitata* bark**


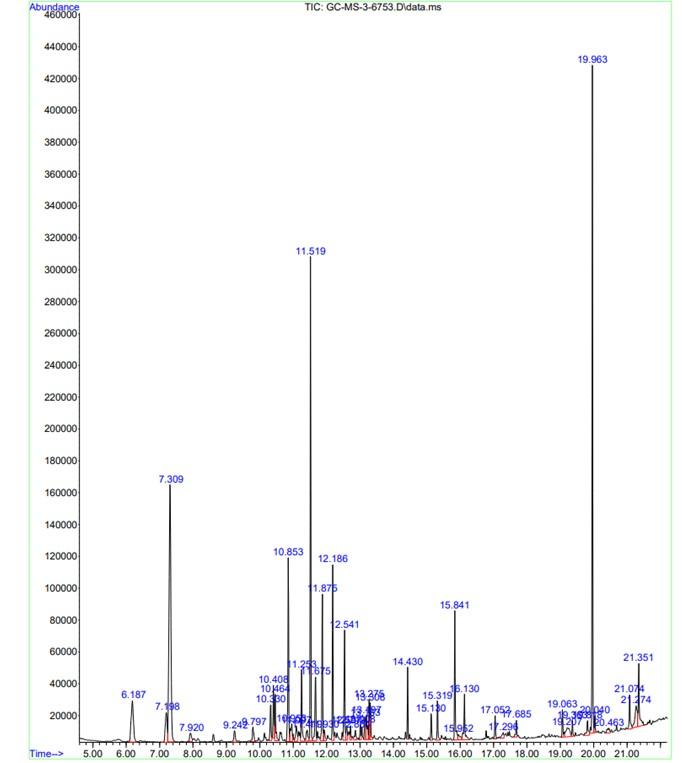


| **Peak (Wave number cm^-1^)** | **Functional group** | **Structural Characteristics** | **Phytocompounds** |
| --- | --- | --- | --- |
| 1617 | Carbonyl group (C=O or CHO) or NH 2, | Stretching vibration | Ketone compounds |
| 1315 | Carboxyl group (COOH) | Stretching vibration | Carboxylic acid  compounds |
| 1029 | S=O Silica group, Phosphate  ion | Stretching vibration | Silicon compound,  Phosphate compound |
| 777 | C-H aromatic, C-Cl stretch | Stretching vibration | Alkanes, Chloro  compounds |
| 657 | C-Br Aliphatic | Stretching vibration | Bromo compounds |
| 3521 | O-H hydroxyl group | Low stretch | Poly Hydroxy compound |

**Supplementary Table 1: FTIR Interpretation of phytocompounds of ethanol extract of *Adansonia digitata* bark**

**Supplementary Table 2: GC-MS Chromatogram of ethanol extract of *Adansonia digitata* bark**

| **S. No** | **Retention time** | **Compound name** | **Molecular formula** | **Mol.Wt** | **% Peak area** |
| --- | --- | --- | --- | --- | --- |
|  | 19.963 | 2H-1,4-Benzoxazine-6-carboxamide, N-cyclopropyl-3,4-dihydro-3-oxo- | C_12_H_12_N_2_O_3_ | 232.23 | 13.86 |
|  |  | Benzyldiethyl-(2,6-xylylcarbamoylmethyl)-ammonium benzoate | C_28_H_34_N_2_O_3_ | 446.6 |  |
|  |  | Furan, 2,2'-methylenebis[5-methyl- | C_11_H_12_O_2_ | 176.21 |  |
|  | 11.519 | (+)-Epi-bicyclosesquiphellandrene | C_15_H_24_ | 204.35 | 10.49 |
|  |  | 1,6-Cyclodecadiene, 1-methyl-5-methylene-8-(1-methylethyl)-, [s-(E,E)]- | C_15_H_24_ | 204.35 |  |
|  |  | 1H-Cyclopenta[1,3]cyclopropa[1,2]benzene, octahydro-7-methyl-3-methylene-4-(1-methylethyl)-, [3aS-(3a.alpha.,3b.beta.,4.beta.,7.alpha.,7aS)]- | C_15_H_24_ | 204.35 |  |
|  | 7.309 | Cyclohexene, 3,4-diethenyl-3-methyl- | C_11_H_16_ | 148.24 | 15.41 |
|  |  | Cyclohexene, 5,6-diethenyl-1-methyl- | C_11_H_16_ | 148.24 |  |
|  |  | Cyclopentane, 1,3-bis(methylene)- | C_7_H_10_ | 94.15 |  |
|  | 10.853 | Bicyclo[5.2.0]nonane, 2-methylene-4,8,8-trimethyl-4-vinyl- | C_15_H_24_ | 204.35 | 5.08 |
|  |  | Caryophyllene | C_15_H_24_ | 204.35 |  |
|  | 12.186 | Cyclohexanemethanol, 4-ethenyl-.alpha.,.alpha.,4-trimethyl-3-(1-methylethenyl)-, [1R-(1.alpha.,3.alpha.,4.beta.)]- | C_15_H_26_O | 222.37 | 3.86 |
|  | 11.875 | Naphthalene, 1,2,3,5,6,8a-hexahydro-4,7-dimethyl-1-(1-methylethyl)-, (1S-cis)- | C_15_H_24_ | 204.35 | 3.64 |
|  | 15.841 | Dibutyl phthalate | C_16_H_22_O_4_ | 278.34 | 3.21 |
|  |  | Phthalic acid, 6-ethyl-3-octyl butyl ester | C_22_H_34_O_4_ | 362.5 |  |
|  | 12.541 | 1-Heptadecene | C_17_H_34_ | 238.5 | 3.07 |
|  |  | 1-Hexadecene | C_16_H_32_ | 224.42 |  |
|  | 14.430 | 1-Nonadecene | C_19_H_38_ | 266.5 | 1.44 |
|  |  | 5-Octadecene, (E)- | C_18_H_36_ | 252.5 |  |
|  |  | 9-Eicosene, (E)- | C_20_H_40_ | 280.5 |  |
|  | 11.253 | alpha-Caryophyllene | C_15_H_24_ | 204.35 | 2.02 |
|  | 11.675 | 1H-Cycloprop[e]azulene, 1a,2,3,5,6,7,7a,7b-octahydro-1,1,4,7-tetramethyl-, [1aR-(1a.alpha.,7.alpha.,7a.beta.,7b.alpha.)]- | C_15_H_24_ | 204.35 | 2.24 |
|  |  | Bicyclogermacrene | C_15_H_24_ | 204.35 |  |
|  | 21.351 | 3-(3,5-Dimethyl-1H-pyrozol-1-yl)-6-(4-morpholinyl)-1,2,4,5-tetrazine | C_11_H_15_N_7_O | 262. | 2.10 |
|  |  | 6-Methyl-cyclohex-2-en-1-ol | C_7_H_12_O | 112.17 |  |
|  |  | Acetic acid, [4-(4-methyl-1-piperazinylcarbonyl)phenyl] ester | C_14_H_18_N_2_O  _3_ | 261.279 |  |
|  | 22.273 | Eicosane | C_20_H_42_ | 282.5 | 1.22 |
|  |  | Heneicosane | C_2_1H_44_ | 296.6 |  |
|  |  | Octadecane | C_18_H_38_ | 254.5 |  |
|  | 10.408 | 7-Hexadecene, (Z)- | C_16_H_32_ | 224.42 | 1.45 |
|  |  | Cyclododecane | C_12_H_24_ | 168.32 |  |
|  | 21.074 | Eicosane, 9-octyl- | C_28_H_58_ | 394.8 | 1.16 |
|  |  | Eicosane | C_20_H_42_ | 282.5 |  |
|  | 15.319 | 2-Butyl-3-(4-.beta.-diethylaminoethoxybenzoyl)benzofuran | C_25_H_31_NO_3_ | 393.5 | 1.10 |
|  |  | 4-Methoxy-2-nitro-N-[.beta.-diethylaminoethyl]aniline | C_13_H_2_1N_3_O_3_ | 267.32 |  |
|  |  | Acetamide, 2-diethylamino-N-[2-(4-hydroxyphenyl)-1,1-dimethylethyl]- | C_16_H_26_N_2_O_2_ | 278.39 |  |
